# Lebenswelten Kitas und Schulen – Herausforderungen für die Gesundheitsämter in der Pandemie

**DOI:** 10.1007/s00103-021-03304-1

**Published:** 2021-04-19

**Authors:** Ulrike Horacek, Isabel Auer, Heidrun Thaiss

**Affiliations:** 1 Ministerium für Arbeit, Gesundheit und Soziales Nordrhein-Westfalen, Fürstenwall 25, Düsseldorf, Deutschland; 2 Bundeszentrale für gesundheitliche Aufklärung (BZgA), Köln, Deutschland

**Keywords:** Kommunaler Öffentlicher Gesundheitsdienst/Gesundheitsamt, Kinder- und Jugendgesundheitsdienst, Kernaufgaben, Kinderbetreuung und Schule, Corona, Local public health service/health authority, Child and youth health service, Core values, Daycare and school, Corona

## Abstract

Kinder- und Jugendgesundheitsdienste (KJGD) als eine kommunale Struktureinheit des deutschen öffentlichen Gesundheitssystems sind mit der Gesundheitsfürsorge für Kinder, Jugendliche und Familien befasst. Wesentliche Aufgaben sind in den Gesundheitsdienstgesetzen der Bundesländer festgelegt und umfassen Tätigkeiten in, für und mit Betreuungs- und Bildungseinrichtungen. Seit Beginn der COVID-19-Pandemie haben sich die Tätigkeiten der KJGD deutlich verändert. In dem vorliegenden Beitrag werden die Aufgaben während der Pandemie dargestellt. Grundlage ist eine Befragung von KJGD-Leitungen in 11 Kommunen im Bundesland Nordrhein-Westfalen im Oktober 2020.

Der KJGD wird während der Pandemie stark in den Infektionsschutz in Kitas und Schulen eingebunden. Dazu gehören Kontaktpersonennachverfolgung, Abstrichentnahme, Beratung, Teilnahme am Krisenstab, Quarantäneüberwachung und Datenerfassung. Originäre Tätigkeiten müssen in vielen Bereichen vollkommen eingestellt oder zumindest stark eingeschränkt werden. Betroffen sind u. a. die Aufgabenbereiche Betriebsmedizin, Gutachten, Schuleingangsuntersuchungen, Feststellung sonderpädagogischer Unterstützungsbedarfe, Gesundheitsberichterstattung, Kooperationen mit Kinderschutz und Frühen Hilfen, Ausbruchsmanagement für andere Erkrankungen und Schließung von Impflücken.

Die Folgen des Aussetzens originärer Tätigkeiten sind noch nicht abzusehen, so z. B. jene von fehlenden Schuleingangsuntersuchungen. Die Wiederaufnahme der Tätigkeiten ist dringend erforderlich. Im Interesse der kommunalen Daseinsvorsorge ist der KJGD ein unverzichtbarer Health-in-all-policies-Akteur, Koordinator und Garant dafür, dass Kinder und Jugendliche nicht nur physisch gesund und von Infektionen verschont bleiben, sondern auch in ihrer Entwicklung und Teilhabe unterstützt werden.

## Abstract (en)

Local public health services in Germany usually include a local organizational unit that is in charge of the healthcare of children, youths, and families (KJGD). Major tasks are defined in the federal laws for the public health service emphasizing different types of activities in daycare units, schools, and community living environments. Since the beginning of the COVID-19 pandemic, the activities of the KJGD have clearly changed. This article will provide a review of these tasks during the pandemic on the basis of interviews with KJGD administrations in 11 communities of the federal state North Rhine-Westphalia in October 2020.

Typically, the KJGD is deeply involved in infection prevention in daycare facilities and schools by providing contact tracing, swab collection, guidance, crisis committee participation, quarantine survey, and data capture. The classic tasks had to be entirely discontinued in some areas, or at least strongly limited. The affected areas include occupational medicine, medical reports, school entry examinations for children, assessment of special education needs, health reporting, cooperation with child protection and early intervention, outbreak management for other infectious diseases, and the closing of immunization gaps.

The consequences of discontinuing the original tasks, e.g., missing school entry examinations, cannot be predicted; resumption of these activities is essential. On behalf of municipal services of general interest in the sense of public health (in all policies), the KJGD makes a remarkable contribution to ensuring that children and youths not only stay physically healthy and far from infections but are also promoted in their individual development and participation.

## Einleitung

**Figure 1:**
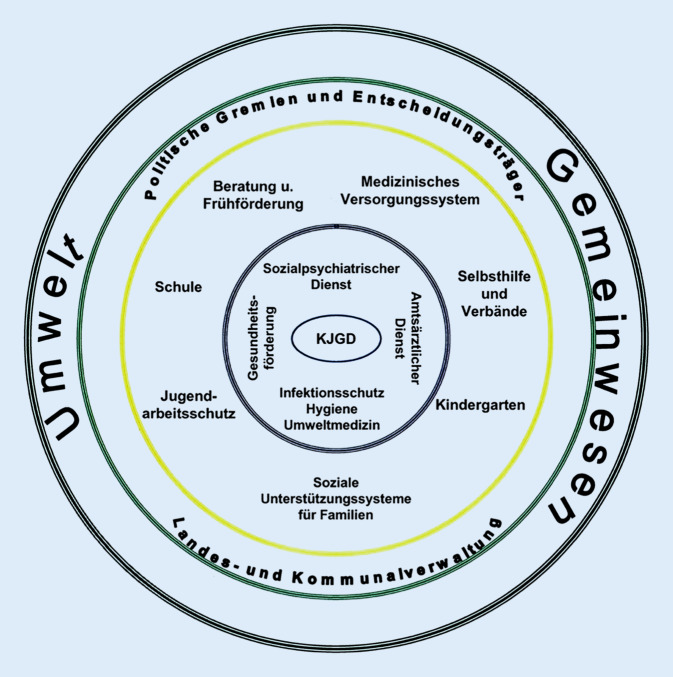

Kinder- und Jugendärztliche Dienste (KJGD, synonym: Fachdienste für Kinder- und Jugendgesundheit) sind neben den Amtsärztlichen und den Sozialpsychiatrischen Diensten sowie dem Infektionsschutz ein Kernbereich des kommunalen Öffentlichen Gesundheitsdienstes (ÖGD) in Deutschland (Abb. 1). Aufgaben des KJGD sind es, die Gesundheit von Kindern und Jugendlichen zu schützen und zu fördern sowie allgemeine und individuelle Gesundheitsgefährdungen zu erkennen, zu mildern oder zu beseitigen. Konkreter umfassen diese Aufgaben [1]:

1. die Gesundheitsförderung in Gemeinschaftseinrichtungen für Kinder,
2. das Hinwirken auf eine gesunde, altersgerechte Entwicklung durch die Feststellung der individuellen Förderbedarfe mittels zielgerichteter Vorsorge- und Früherkennungsuntersuchungen bei Kindern und Jugendlichen in Kindergärten und Schulen,
3. die Reduzierung der Folgeschäden bei Kindern und Jugendlichen mit Entwicklungsstörungen und Behinderungen durch sozialpädiatrische Hilfen (auch aufsuchend) sowie
4. die Beratung der öffentlichen Entscheidungsträger unter anderem in Form der Gesundheits-(und Sozial‑)Berichterstattung.

Das Anliegen, die Gesundheit von Kindern und Jugendlichen zu erhalten, soll durch die Gesundheitserziehung, das Impfwesen sowie Aufklärungs- und Beratungsangebote bei Reihenuntersuchungen (Schuleingang) oder in Sprechstunden für Schüler, Eltern, pädagogische Fachkräfte und gegebenenfalls weitere Bezugspersonen erreicht werden. Bei Entwicklungsverzögerungen beziehungsweise Behinderungen bietet der KJGD verschiedene Hilfen nach den Sozialgesetzbüchern (SGB V, SGB VIII, SGB IX, SGB XII ff.) an, die sowohl in den Fachdiensten selbst als auch auf deren Vermittlung hin von anderen Einrichtungen erbracht werden. Hierzu gehören zum Beispiel Frühförderung, Eingliederungshilfe oder Sonderpädagogik [2].

In den ÖGD-Gesetzen vieler Bundesländer wird diesem explizit eine betriebsmedizinische Verantwortung zugeschrieben, die sich auf Kinder bzw. Schüler in Gemeinschaftseinrichtungen bezieht. Für den „Arbeitsplatz Schule“ wird sie von einem Schularzt und seinem Team umgesetzt. Hier geht es zum einen um Individualuntersuchungen und die Klärung von Fragen zu Unterstützungsbedarfen im Kita- und Schulalltag (zu denen auch viele Aspekte der Schuleingangsuntersuchungen zählen), um die gutachterliche Tätigkeit im Kontext chronischer Erkrankungen, um Fragen der Inklusion, um die gemeinwesenbezogene Zusammenarbeit mit Jugend- und Sozialhilfe und vieles mehr. Zum anderen geht es aber auch um die Gestaltung gesundheitsförderlicher Rahmenbedingungen, um ein kleinräumiges Monitoring des Gesundheitszustands, die Kommunikation darüber und das Ableiten von Handlungsempfehlungen und Maßnahmen für die Verantwortungsträger sowie um Politikberatung. Diese Aufgaben sind von ihrer Natur in der kommunalen Daseinsvor- und -fürsorge verankert. Sie werden in der Regel von einem multiprofessionell aufgestellten KJGD erfüllt und haben einige natürliche Schnittmengen mit den Aufgaben, die im Ressort für Öffentliche Hygiene und Infektionsschutz des Gesundheitsamts zu erfüllen sind.

Die regelhaften Leistungen der KJGDs, zumeist in den Gesundheitsdienstgesetzen der Länder oder kommunalen Verordnungen festgelegt, haben nun durch den Beginn der COVID-19-Pandemie einen jähen Stopp erfahren. Die wichtigen Einrichtungen von Betreuung und Bildung für Kinder sind besonders betroffen, treffen doch hier verschiedenste institutionelle Verpflichtungen und Aufträge auf unterschiedliche Ansprüche, Erwartungen und Interessen. Es ist für einen Großteil der Bevölkerung – alle Familien mit Kindern – wichtig, dass der Betrieb von Kita, Schule oder Hort verantwortungsbewusst und vertretbar gestaltet wird. An der Basis, in der Kommune vor Ort, musste und muss sich der Öffentliche Gesundheitsdienst auch hier bewähren – nicht nur in der Erfüllung von Aufgaben, die in direktem Bezug zu Fall- und Verdachtsfällen einer SARS-CoV-2-Infektion, zur Kontaktpersonennachverfolgung oder gar zum Ausbruchsmanagement stehen.

Es soll deshalb der Frage nachgegangen werden, welche Aufgaben zur Pandemiebewältigung der KJGD inner- oder außerhalb von Bildungseinrichtungen konkret übernommen hat, welche Aufgaben stattdessen teilweise oder gänzlich entfallen sind und welche mittel- und langfristigen Folgen dadurch erwartet werden können.

## Befragung von KJGD-Leitungen

Im bevölkerungsreichsten Bundesland Nordrhein-Westfalen (NRW) wurde im Oktober 2020 ein Survey mit 4 standardisierten offenen Fragen zur aktuellen Situation in Form einer repräsentativen Stichprobe bei Leitungen der KJGD durchgeführt. Es wurden 11 Kommunen mit heterogener Bevölkerungszahl und -struktur [3] erfasst, die eine Einwohnerzahl von insgesamt 3,7 Mio. haben und damit mehr als 20 % der NRW-Bevölkerung repräsentieren [4]. Bezogen auf die Einschulungszahlen im Schuljahr 2019/2020 weist die amtliche Statistik des Landes im Bildungsportal 679.109 einzuschulende Kinder aus, die befragten Kommunen sind für knapp 20 % verantwortlich. Die Befragung erfolgte schriftlich und wurde bei Bedarf durch telefonische Nachfragen ergänzt. Alle adressierten KJGD-Leitungen haben teilgenommen.

Folgende Fragen wurden gestellt:

1. Wie sind Sie in die pandemiebedingten Aufgaben des Infektionsschutzes (Kontaktnachverfolgung, Abstrichentnahme, telefonische Beratung, Mitwirkung in Steuerungsgremien etc.) eingebunden/abgeordnet?
2. Üben Sie fachlich beratende Aufgaben in Schulen und Kitas aus?
3. Welche Auswirkungen hat die Pandemie auf Ihre originären gesetzlichen (und sonstigen) Aufgaben?
4. Was wünschen Sie sich aktuell und langfristig am meisten?

## Ergebnisse der Befragung

### Aufgaben im Infektionsschutz

In allen befragten Ämtern waren die KJGD-Teams mit pandemiebezogenen (ressortfremden) Aufgaben betraut (Tab. 1). In die Kontaktpersonennachverfolgung wurden ausnahmslos alle KJGDs eingebunden, desgleichen in die telefonische individuelle Beratung von Indexpersonen, Kontaktpersonen und Menschen in Quarantäne. Sozialmedizinische Assistentinnen haben vielfach die Hotline übernommen.

**Table 1:** 

Kommune Nr.	Kontaktpersonennachverfolgung	Abstrichentnahme	Beratung (telefonisch/persönlich)	Teilnahme Krisenstab/Steuerungsgremium	Quarantäneüberwachung	Datenerfassung/Dokumentation	Sonstiges/Bemerkungen/spezielle Aufgaben
1	X	X	X	X	X	X	Einsatz in mobilen Testteams Koordination der Labore
2	X	0	X	(X)	X	X	Übernahme nichtärztlicher Aufgaben, z. B. Anfordern von Schülerlisten
3	(X)	0	(X)	0	(X)	X	Alle Berufsgruppen des KJGD komplett eingebunden
4	X	X	X	0	X	(X)	Ärztliche individuelle Beratung von Kontaktpers. und Quarantänisierten (Tagebuch)
5	X	0	X	X	X	(X)	Entlassmanagement aus stationärer Behandlung
6	X	0	X	X	X	(X)	Schulung des Unterstützungspersonals
7	X	X	X	X	(X)	(X)	Weiterbeschäftigung ärztlichen Personals über Ruhestand hinaus
8	X	(x)	X	0	(X)	(X)	Seit Frühjahr fast kompletter Einsatz des Teams im Infektionsschutz; Beratung zu Hygienekonzepten in Kitas und Schulen
9	X	0	X	X	X	(X)	Übernahme aller Fälle in Kitas und Schulen; Einrichtungsberatung in diesem Kontext
10	X	0	X	(X)	X	(X)	Terminvergabe für Abstrichzentren Untersuchungen bei „Maskenverweigerern“
11	X	0	X	X	(X)	0	Übernahme der Gesundheitsamtsleitung während Pandemie

Legende:

*X* Aufgabe wurde übernommen

*(X)* Aufgabe wurde teilweise oder zeitweise übernommen

*0* Aufgabe wurde nicht übernommen

Das Robert Koch-Institut (RKI) hat das Vorgehen in der Fallverfolgung in einem Flussschema dargestellt und geschätzte Zeitaufwände für die einzelnen Schritte angegeben. Demnach sei mit ca. 55 min für Informationsaufnahme und Ermittlung im Kontext des ersten Fallkontakts, 8 min für die Fallverfolgung pro Tag und ca. 25 min für die Kontaktpersonenverfolgung pro Kontakt zu rechnen. Da mittlerweile die Zahl der Kontaktpersonen häufig sogar im dreistelligen Bereich liegt, lässt sich die zeitliche Beanspruchung abschätzen. Als wesentlich erwies sich nach Auskunft der Befragten die Notwendigkeit, eine ärztliche Ansprechperson für Erkrankte vorzuhalten.

Operative Unterstützung erfolgte in der personellen Besetzung von festen oder mobilen kommunalen Testzentren, aber auch koordinierend in deren Aufbau, im Kontakt mit den Laboren, bei Schulungen des Einsatzpersonals und Weiterem. Mehrere KJGD-Leitungen waren fester Bestandteil des Krisenstabs oder anderer kommunaler Steuerungsgremien und haben in der Pandemiesituation die stellvertretende oder kommissarische Gesundheitsamtsleitung übernommen.

Bezogen auf Gemeinschaftseinrichtungen wie Kita und Schule, aber auch Nachmittagsbetreuung waren die Ärzte beratend tätig, um das Management von Index- oder Kontaktpersonen konform mit den RKI-Empfehlungen abzustimmen. Hier wurde das Ziel verfolgt, vertretbare Lösungen zu finden, um umfangreiche Schließungen zu vermeiden; dazu waren häufig sehr zeitaufwändige und anspruchsvolle Ermittlungen zu Zeitpunkt, Dauer und Art der Kontakte erforderlich.

### Beratende Aufgaben in Kitas und Schulen

Aus den Antworten auf die zweite Frage nach spezifischen Beratungsaufgaben in Schulen und Kitas ging hervor, dass diese Leistungen vom KJGD durchweg einen großen Raum einnahmen. Vielfach wurden Fragen der Ortshygiene komplett vom Infektionsschutzressort auf den KJGD übertragen, da dieser durch seine betriebsmedizinische Tätigkeit zumeist über langjährige Kenntnisse der Settings und verlässliche Kooperationsstrukturen verfügt. Es galt, Hygienekonzepte, basierend auf den bestehenden Rahmenhygieneplänen, um erregerspezifische Aspekte ortskonform zu erweitern bzw. Anpassungen vorzunehmen; hierbei zeigte sich ein erheblicher Beratungs- und Abstimmungsbedarf. Bezogen auf ein einzelnes Amt wird das Ausmaß der Beratungsleistungen vorstellbar, wenn man die Anzahl der Schulen in NRW mit derzeit 2793 Grundschulen und fast ebenso vielen Schulen anderer Schultypen in Betracht zieht, die sich auf die Zuständigkeit von 53 Gesundheitsämtern verteilt.

Hinzu kommt das – für den KJGD alltägliche – Erfordernis, mit zahlreichen Adressaten und Berufsgruppen in unterschiedlichsten Funktionen und Rollen zielorientiert zusammenzuwirken. Beispiele sind Schul- und Kitaträger, Erzieher, Kitaalltagshelfer, Lehrer, Integrationshelfer, Schulsozialarbeiter, Schulpsychologen, Schulbegleiter, Schulküchenpersonal, Hausmeister, Fahrer des Schülerspezialverkehrs, Personal des Offenen Ganztags, Unterstützungspersonal in Kitas, Tagesmütter, ehrenamtliche Helfer u. v. a. m.

Mit Fortschreiten der SARS-CoV-2-Ausbreitung wurden Aufgaben der Fallverfolgung und des Ausbruchsmanagements zunehmend auf alle Berufsgruppen und Mitarbeiter der KJGD übertragen; so kamen auch (zahn-)medizinische Fachangestellte, sozialmedizinische Assistenten und Zahnärzte zum Einsatz.

Die nachfolgende Aufstellung informiert exemplarisch über pandemiebezogene Beratungsinhalte:

#### Notbetreuungsanspruch:

Beim Start der Notbetreuung galt es häufig, individuell zu klären, für welche Kinder mit *relevanten Vorerkrankungen* und/oder mit *vulnerablen Personen in der Haushaltsgemeinschaft* eine Teilnahme an der Notbetreuung zu verantworten war [5, 6], unter Berücksichtigung der geringen Erregerprävalenz in Kitas und des niedrigen Positivenanteils bei getesteten Unter-15-Jährigen zwischen 2,3 % und 3,0 % [7]. An dieser Stelle erweist sich die Expertise des KJGD als hilfreich, da er mit den Settings und dem Umgang mit infektiösen Erregern vertraut ist. Er kann zielgerichtet mit den betreuenden Kinder- und Jugendärzten oder Hausärzten kommunizieren und zu einer fachlich begründeten, individuellen Entscheidung beitragen.

#### Präsenz bei Abschlussprüfungen:

Der Beginn der Pandemie fiel mit den Vorbereitungen für Abitur- und weitere *Abschlussprüfungen *zusammen; hier musste erwogen werden, wie die notwendigen präsenzgebundenen mündlichen Prüfungen „coronakonform“ gestaltet werden konnten.

#### Anpassung von Hygieneplänen:

Im Kontext der Wiederöffnung von Betreuungs- und Schulbetrieb mussten die *Hygienepläne* wieder neu justiert, d. h. auf die neue Situation größerer Kinder- und Schülerzahlen angepasst werden.

#### Erklärungs- und Erläuterungsbedarf von Vorschriften:

Unterstützungsbedarf ergab sich sowohl für Landesregierungen, Schulleitungen, Träger von Einrichtungen wie Eltern und kommunale Amtsträger bei der „Übersetzung“ von Verordnungen und Verwaltungsvorschriften auf die lokale Situation vor Ort [8–10].

#### Koordination ehrenamtlicher Aktivitäten:

Als große Ausbrüche in der Fleisch verarbeitenden Industrie auftraten, unterstützte der kommunale KJGD die Organisation und Koordination von ehrenamtlichen Aktivitäten für die betroffenen Familien und stand diesen in der gesundheitlichen Sorge und Betreuung ihrer Kinder beratend zur Verfügung – eine klassische Situation *subsidiären Zugangs* und Vorgehens.

#### Rechtliche Fragen von Eltern:

Für Eltern ergab sich sehr häufig Bedarf an elementarer *„Rechtsberatung“,* z. B. bezüglich der Ansprüche, die sich aus behördlich ausgesprochener Quarantäne ergaben, zur Möglichkeit von Großelternbesuchen oder dem Einsatz von (vulnerablen) Großeltern in der nachmittäglichen Kinderbetreuung, zu Erschwernissen durch getrennt lebende Elternteile u. a.

#### Abstimmung zur Verpflegung:

Modalitäten des (Weiter‑)Betriebs von *Mensen und Cafeterien*; in den Kitas: Modalitäten der *Essenszubereitung und Mahlzeiteneinnahme* waren häufig unter Moderation des KJGD abzustimmen.

#### Organisation von PCR-Testungen:

Groß war die Nachfrage von Betreuenden und Lehrenden zur möglichst effektiven *Organisation der PCR-Testungen*. Hierzu wurde z. B. durch den Einsatz einer koordinierenden *Schulgesundheitsfachkraft *in einem großen Schulzentrum eine beispielgebende Lösung gefunden, mit der die Zeit bis zur Ergebnisübermittlung verkürzt werden konnte.

#### Zähneputzen in Kitas:

Nicht selten wurde aus den Betreuungseinrichtungen die Frage an den KJGD gestellt, ob denn das regelmäßig praktizierte, eingeübte* Zähneputzen* nach den Mahlzeiten weiter erfolgen könne. Grundsätzlich konnten hier – in Abhängigkeit von den räumlichen und personellen Rahmenbedingungen – infektionsschutzkonforme Vorgehensweisen gefunden werden.

#### Belüftung von Räumlichkeiten:

Im Verlauf der Pandemie rückten spezifische Fragen zur Belüftungssituation von vorwiegend schulischen Räumlichkeiten in den Vordergrund [11], die häufig nur konkret durch Inaugenscheinnahme von Belüftungsmöglichkeiten angemessen beantwortet und gelöst werden konnten. Hier ging es nicht nur um Unterrichtsräume, sondern z. B. auch um Sporthallen und Schwimmbäder mit schulfremder Nutzung.

#### Schülerspezialverkehr:

Inklusiv beschulte Schüler oder solche, die spezielle Förderschultypen besuchen, werden häufig in Kleinbussen mittels *„Schülerspezialverkehr*“ transportiert. In dieser Gruppe ist die Zahl der Kinder hoch, die aus gesundheitlichen Gründen keine Alltagsmaske tragen können und dadurch andere Mitfahrende, aber auch sich selbst gefährden können. Hier galt es, vertretbare Lösungen auch im Sinne des Arbeitsschutzes für das Fahrpersonal zu finden.

#### Bewertung respiratorischer Symptome:

Dominierendes Beratungsthema war jedoch die *Bewertung respiratorischer Symptome*. Zu dieser Thematik existieren mehrere Stellungnahmen von Fachgesellschaften [12–15], deren Empfehlungen weitgehend in die entsprechenden Handreichungen der Länder für Gemeinschaftseinrichtungen [5, 6, 16, 17] aufgenommen wurden. Hier war meist die Einordnung der Häufigkeit von Infekten im Kleinkindalter, insbesondere in Krippe und Kita, auch vor dem Hintergrund der zunehmenden epidemiologischen Erkenntnisse zur Übertragungswahrscheinlichkeit durch Kinder, andererseits unter Berücksichtigung des individuellen und einrichtungsbezogenen Risikos vorzunehmen [12–15]. Daneben galt es oft klarzustellen, dass für Impf‑, Infektions- und Arbeitsschutz der Beschäftigten in Gemeinschaftseinrichtungen (Erzieher, Lehrer) primär der Dienstherr bzw. Anstellungsträger verantwortlich ist.

### Auswirkungen der Pandemie auf originäre Aufgaben

Die dritte Frage zielte darauf ab, welche originären KJGD-Aufgaben während der Pandemie ganz oder teilweise entfallen mussten; die Antworten sind in Tab. 2 dargestellt. Erläuternd ist zu ergänzen, dass im Bereich des Gutachtenwesens sowohl die Fragestellungen fokussiert und oft auch zugunsten neuer Problematiken verschoben werden mussten (z. B. Stellungnahmen nach Aktenlage, Basisscreening Hören/Sehen/Impfstatus anstelle von standardisierten Schuleingangsuntersuchungen (SEU) etc.).

**Table 2:** 

Kommune Nr.	1	2	3	4	5	6	7	8	9	10	11
Betriebsmedizinische Aufgaben in Gemeinschaftseinrichtungen für Kinder und Jugendliche	0	0	0	0	0	0	0	0	0	0	0
Gutachterliche Tätigkeit	(X)	(X)	(X)	(X)	(X)	(X)	(X)	(X)	(X)	(X)	(X)
Schuleingangsuntersuchungen (SEU)	0	(X)	(X)	(X)	0	0	(X)	0	(X)	(X)	(X)
Kontext sonderpädagogischer Unterstützungsbedarf (AO - SF)	(X)	(X)	(X)	(X)	(X)	(X)	(X)	(X)	(X)	(X)	X
Beratung von Kitas und Schulen	X	0	0	0	0	0	0	(X)	0	(X)	0
Gesundheitsberichterstattung, Epidemiologie	0	0	0	0	0	0	0	0	0	0	0
Zielgruppen- und bedarfsorientierte Untersuchungen	0	0	0	(X)	(X)	(X)	(X)	(X)	0	X	X
Kooperation Kinderschutz und Frühe Hilfen	0	0	0	0	0	(X)	0	(X)	(X)	X	(X)
Ausbruchsmanagement in Gemeinschaftseinrichtungen für Kinder (außer COVID-19)	(X)	(X)	(X)	0	X	0	0	0	0	0	0
Maßnahmen zum Schließen von Impflücken	0	0	0	0	0	0	0	0	0	0	0
Beratung der Kommune/Gesundheitsplanung mit Sozialraumbezug	0	0	0	0	0	0	0	0	0	0	0

Legende:

*X* Aufgabe wurde wahrgenommen

*(X)* Aufgabe wurde teilweise oder zeitweise oder mit reduziertem Standard wahrgenommen

*0* Aufgabe wurde nicht wahrgenommen

Neue Fragestellungen waren z. B. Untersuchungen von „Maskenverweigerern“ mit nicht aussagekräftigen Freistellungsattesten sowie Anträge auf Befreiung vom Präsenzunterricht. Im Hinblick auf eine möglichst weitgehende Integration wurde eine Vielzahl von Kindern vorgestellt, welche sich mit der pandemiebedingten Situation aufgrund gesundheitlicher Beeinträchtigungen nur schwer arrangieren konnten und für die gemeinsam nach geeigneten Rahmenbedingungen gesucht wurde, sowie Kinder, für die Unterstützungs- und Leistungsanträge auf den Weg gebracht werden mussten.

Schuleingangsuntersuchungen erfolgten im Umfang zwischen 10 % und ca. 80 % (z. T. als Screening, nicht den Landesstandards entsprechend). Allerdings wurden bei Kindern, bei denen gesundheitliche Beeinträchtigungen bekannt und/oder bei denen sonderpädagogische Förderbedarfe absehbar waren, fast ausnahmslos schulärztliche Gutachten im Rahmen der Ausbildungsordnung Sonderpädagogische Förderung durchgeführt.

### Aktuelle und langfristige Wünsche der KJGD-Leitungen

Die vierte Frage zielte auf die drängendsten aktuellen Probleme der KJGD-Leitungen ab.

Einhellig wurde von allen Befragten der dringende Wunsch geäußert, wieder zur eigentlichen Rolle im System der öffentlichen Kindergesundheit und damit zum KJGD-Kernaufgabenspektrum zurückkehren zu können. Expertise, Erfahrung und Neigung sind hier in besonderer Weise vorhanden. Dabei wurde auch das Erfordernis gesehen, den Bereich Infektionsschutz mit eigenen, klar zugeordneten Fach- und Unterstützungskräften weiter und nachhaltig auszubauen.

Die Rückkehr zum originären Aufgabenbereich wurde nicht nur an erster Stelle genannt, weil das Arbeiten im vertrauten Bereich persönlich als besonders erfüllend erlebt wird; es wurde ein dem Bedürfnis entsprechender Bedarf reklamiert, da für die Kinder, Jugendlichen und Familien Nachteile zu befürchten seien, wenn diese Tätigkeiten auch mittel- bis langfristig entfielen. Konkret wurde in den ergänzenden telefonischen Interviews auf die Notwendigkeit hingewiesen, z. B. Impflückenschließungsprogramme und Schulsprechstunden in Brennpunktschulen wiederaufzunehmen.

Es wurde aus der Außensicht berichtet, dass Schulen die vertiefte gutachtenbezogene schulärztliche Tätigkeit vornehmlich für Kinder mit sonderpädagogischem oder gesundheitsbezogenem Unterstützungsbedarf vermissen würden; Jugendämter reklamierten für weniger, aber gravierende Kinderschutzfälle die bewährte Kooperation, und Kitas hätten gern wieder auf die KJGD-Expertise bei Kindern mit verschiedensten Entwicklungsauffälligkeiten zurückgreifen wollen. Um Folgeschäden zu vermeiden, müsse man genau diesen Jahrgang besonders im Blick behalten und sei in besonderer Sorge um psychische Langzeitfolgen der Pandemie.

In der Sorge um das Fortbestehen der Attraktivität des Ressorts KJGD, sollte er denn längerfristig fachfremde Aufgaben übernehmen müssen, wünschte man sich Erfolg bei der Suche nach Fachkräftenachwuchs, befördert durch entsprechende Finanzierungsregelungen.

Wertschätzende Anerkennung des umfänglichen, flexiblen und engagierten „selbstlosen“ Einsatzes während der Pandemie wäre willkommen – anstatt der aktuellen Sorge, dass vorübergehend weniger prioritäre Aufgaben ganz aus dem Blickfeld geraten und qualitativ oder existenziell gefährdet sind.

## Diskussion

### Fehlende Schuleingangsuntersuchungen haben Konsequenzen

Die Befragungsergebnisse verdeutlichen, in welch großem Umfang der KJGD in pandemiebezogene Aufgaben des Infektionsschutzes eingebunden war und zum großen Teil noch ist. Was bedeutet nun ein Wegfall der originären Aufgaben des KJGD konkret für die betroffenen Kinder, ihre Familien und die Kommune? Dies kann exemplarisch an den ausgesetzten oder nur zu einem Drittel und selektiv durchgeführten Schuleingangsuntersuchungen (SEU) dargestellt werden.

Die individualmedizinische Bedeutung der SEU bezieht sich auf die Beurteilung der körperlichen, geistigen, sozialen und emotionalen Entwicklung des angehenden Schulkinds. Dabei wird untersucht, ob das Kind den Anforderungen des Schulalltags aus medizinischer Sicht gewachsen ist (körperliche/geistige Beeinträchtigungen, Hinweise auf Verhaltensauffälligkeiten o. Ä.). Welche Erkrankungen, spezielle Förderbedarfe oder Entwicklungsauffälligkeiten liegen vor, und kann diesen (ggf. schon vor Eintritt in die Schule) begegnet werden? Gibt es abklärungsbedürftige Befunde? Sind Hilfsmittel, z. B. eine Brille, notwendig? Besitzt das Kind genug Selbstvertrauen, Lernbereitschaft, Konzentrationsfähigkeit, Ausdauer und Frustrationstoleranz, um den Schulalltag zu bewältigen? Welche besonderen Fähigkeiten/Begabungen bringt das Kind mit? Wie können diese vonseiten der Eltern und der Schule unterstützt und gefördert werden? Die Kindervorsorgeuntersuchung U9 (60. bis 64. Monat) mit ihrer eher morbiditätsorientierten Fragestellung kann dabei kein Ersatz für die SEU sein [18].

Aus „arbeitsmedizinischer“ Sicht hat die SEU die Aufgabe, Unterstützungs- und Förderbedarfe des Schulkinds zu identifizieren und geeignete Maßnahmen in die Wege zu leiten. Dies ist auch aus sozialkompensatorischer Perspektive unverzichtbar, um Chancenungleichheiten zu identifizieren und nach Möglichkeit auszuräumen. Aus epidemiologischer Sicht ist es hilfreich, den Gesundheitszustand und die Förderbedarfe jedes Jahrgangs zu erkennen, um daraus Maßnahmen auf kommunaler, idealerweise auch auf Landesebene ableiten zu können.

Fällt die SEU als Screeninguntersuchung aus, können ihre o. g. Funktionen nicht oder nur teilweise erfüllt werden, und der sich daraus ergebende individuelle und settingbezogene Nachteil kann gravierende Folgen haben. Zahlreiche Befunde, die erheblichen Einfluss auf den Schulalltag und den Bildungsweg von Kindern haben und weitergehende Maßnahmen erfordern (Logopädie, Ergotherapie, Heil- und Hilfsmittelversorgung, psychologische Beratung, Differenzialdiagnostik etc.), werden bei den flächendeckenden SEU oft erstmalig festgestellt.

Zum Beispiel traten in Schleswig-Holstein bei den im Schuljahr 2017/2018 einzuschulenden 22.898 Kindern [19] verschiedene Auffälligkeiten stark gehäuft auf: Bei einem Viertel der Kinder (28,0 %) lagen *Sprachauffälligkeiten* vor, ein Sechstel der Kinder befand sich in logopädischer Behandlung oder bedurfte einer solchen (15,9 %). *Verhaltensauffälligkeiten* traten ebenfalls sehr häufig auf (21,4 %). Letztere beeinflussen sowohl den Schulalltag als auch Gesundheits- und Bildungschancen in besonderem Maße. Bei den *Entwicklungsauffälligkeiten* war vor allem die *Körperkoordination* (21,5 %) betroffen. 20,0 % aller Jungen und 13,8 % aller Mädchen der angehenden Schulkinder hatten Schwierigkeiten in der visuellen Wahrnehmung und beim logischen Schlussfolgern. Es besteht ein Zusammenhang zwischen sozialer Lage der Familie und Verhaltens- und Entwicklungsbeeinträchtigungen des Kindes. Zu spät erkannte schulrelevante Auffälligkeiten oder Entwicklungsverzögerungen bergen Risiken in Hinblick auf dauerhafte Sprachentwicklungsstörungen, Lese- und Rechtschreibschwierigkeiten, Lernprobleme sowie emotionale und soziale Probleme. Die SEU sind daher unverzichtbar.

Bei 13.076 im Jahr 2015 in Köln untersuchten Kindern zeigte sich, dass 26 % eine chronische Erkrankung oder Entwicklungsverzögerung aufwiesen; eine Identifikation des Förderbedarfs allein mithilfe eines Screeningfragebogens (Children with Special Health Care Needs) gelang nur bei 6 % der untersuchten Kinder. Dies unterstreicht den Stellenwert der persönlichen Untersuchung durch qualifiziertes, erfahrenes Personal [20].

### Familiäre Unterstützungsbedarfe müssen rechtzeitig erkannt werden

Es offenbaren sich also Problemlagen von häuslicher Gewalt, von Sucht- oder Drogenproblematiken oder anderweitig belasteten, hilfebedürftigen Eltern und Erziehungsberechtigten; es stellen sich Fragen zur Inklusion chronisch kranker Kinder in Kita und Schule sowie zur Integration von Kindern aus anderen Sprach- oder Kulturkreisen: Hierfür ist der ÖGD und insbesondere der KJGD, zumeist in Kooperation mit den sozialpsychiatrischen oder amtsärztlichen Fachbereichen, gerade in Kita und Schule mit niedrigschwelligem Zugang für alle Kinder wertvoller Ansprechpartner und Weichensteller.

### Der KJGD hat eine eigene Rolle in der kommunalen Daseinsfürsorge

Dass zum Schutz der Bevölkerung bei einer epidemischen Lage von nationaler Tragweite alles Erforderliche zu unternehmen und bei Bedarf auch Personal aus anderen Bereichen einzusetzen ist, ist unbestritten. Allerdings sollte der Fokus nicht nur auf das infektiologische Geschehen gerichtet werden. Wie die Ergebnisse unterschiedlicher Befragungen wie des COVID-19 Snapshot Monitorings (COSMO [21, 22]) und anderer bevölkerungsweiter Surveys zeigen, bilden sich die Folgen des Infektionsgeschehens nicht nur in Engpässen der medizinischen und pflegerischen Versorgung, in Sterbetafeln und morbiditätsbezogenen Langzeitschäden ab. Insbesondere sind Familien und ihre Kinder – hier besonders Säuglinge, Klein- und Schulkinder – in vielfältiger und komplexer Weise betroffen. In Familien zeigen sich die Auswirkungen des Managements von Infektions‑, Verdachts- oder Kontaktfällen, aber vor allem auch Nebeneffekte von Maßnahmen wie häuslicher Beschulung, sozialen Kontaktbeschränkungen, familiären generationsübergreifenden Besuchsverboten und reglementierten Freizeitaktivitäten wie Spiel und Sport.

Zur Erkennung möglicher Folgen für die Entwicklung von Kindern, für ihre körperliche und seelische Gesundheit und die soziale Integration in ihren Lebenswelten werden die Kinder- und Jugendgesundheitsdienste mit ihrer Public-Health-Expertise und ihren sozialkompensatorischen Möglichkeiten gebraucht – mehr denn je. Im Gemeinwesen stellen sie eine unverzichtbare „Spezialeinheit“ für kommunale Daseins- und Gesundheitsfürsorge dar. Den KJGD ebenso wie die Bereiche des Infektionsschutzes zu stützen, zu stärken und auszubauen, lohnt sich. Es ist eine Investition in die Zukunft von Kindern und Familien und damit nachwachsenden Generationen, in soziale Chancengleichheit und damit letztendlich in den sozialen Frieden unseres Landes.

## Ausblick

Ein ÖGD, der sich als Dienstleister im Gesamtspektrum kommunaler Daseinsfürsorge versteht, wird in der weiteren Pandemieentwicklung sicherlich nicht weniger wertvoll sein als zu Beginn. Dabei ist insbesondere der KJGD systemrelevant bei der Verhinderung langfristiger Pandemiefolgen für die physische und psychische Gesundheit von Kindern und ihren Familien. Er kann aufbauend auf den aktuellen Erfahrungen und synergistisch mit anderen Verantwortlichen – im Sinne von Health in all Policies – zukünftige Bedingungen für Betreuung und Bildung in der Kommune konkret mitgestalten und weiterentwickeln.

In der Rückkehr zur „Normalität“, d. h. Wiederaufnahme der bisherigen Aufgaben, die größtenteils als Pflichtaufgaben und Selbstverwaltungsaufgaben zur Erfüllung nach Weisung begründet sind, liegt die Chance, aus den „lessons learned“ im Sinne der Politikberatung den Blick zu weiten (Infobox 1).

### Infobox 1 Perspektivisch wichtige Erfahrungen und Erkenntnisse aus der Coronapandemie

- Schulgesundheitsfachkräfte können – am sinnvollsten in Anbindung an den KJGD – verschiedene Aufgaben sinnvoll unterstützen, so z. B.
  - als Hygienelotsen der Schule mit verhaltensbezogenen Präventionsmaßnahmen vor Ort,
  - als Frühwarnsystem für Gefährdungen psychischer Gesundheit von Schülern, voraussichtlich in der postpandemischen Phase von besonderer Bedeutung,
  - in der Kooperation mit Jugendhilfe und anderen kommunalen Akteuren zur Unterstützung von Kindern aus benachteiligten Familien.
- Ein Ausbau der Qualifizierungsmöglichkeiten für multiprofessionelle Teams wäre sinnvoll.
- Fachdienstübergreifend und flächendeckend besteht Bedarf an einheitlicher Software für den ÖGD. Dadurch und durch sinnvolle Verknüpfungen kann der Erkenntnisgewinn aus der kommunalen Gesundheitsberichterstattung erweitert werden.
- Gerade in Krisensituationen ist die Bildung von synergistischen Kooperationen mit kommunalen Akteuren vonnöten. Daraus gewonnene Erfahrungen können für die generelle interprofessionelle Zusammenarbeit genutzt werden (sowohl innerhalb des ÖGD wie außerhalb, somit sektorenübergreifend).
- Eine stärkere Präventionsorientierung durch Kooperation mit den kommunalen „Kümmerern“ des GKV-Bündnisses für Gesundheit ist anzustreben.
- Wissenschaftsfundierung und Kooperation mit Hochschulen sollten verstärkt werden.

Frühe Betreuung und Jugendhilfe, Schule und Bildungssystem, Gesundheitsversorgung (ambulant und stationär), öffentliche kommunale Gesundheits- und Daseinsvorsorge werden in einen Prozess der Neujustierung und Neudefinition eintreten. Es bleibt zu hoffen, dass der kommunale ÖGD mit der gegenwärtigen Aufmerksamkeit für den Bereich des Infektionsschutzes und dem diesbezüglichen Erfolg gestärkt aus der Pandemie hervorgeht. Stark bleibt er aber nur, wenn er auch in der postpandemischen Phase all seine Potenziale einzubringen und zu nutzen weiß. Nur wenn er als Gesamtorganismus auftritt, im Zusammenspiel mit all seinen Elementen und Fachdiensten und in Kooperation mit anderen Ressorts auf einem soliden daten- und wissenschaftsbasierten Fundament agiert, wird er auch zukünftig nicht nur „Krisenpolizei“, sondern auch ein attraktiver Arbeitgeber und wirkmächtiger Public-Health-Akteur für die Menschen in der Kommune und darüber hinaus sein können.
