# Erratum zu: Lebenswelten Kitas und Schulen – Herausforderungen für die Gesundheitsämter in der Pandemie

**DOI:** 10.1007/s00103-022-03572-5

**Published:** 2022-08-01

**Authors:** Ulrike Horacek, Isabel Auer, Heidrun Thaiss

**Affiliations:** 1 Ministerium für Arbeit, Gesundheit und Soziales Nordrhein-Westfalen, Fürstenwall 25, Düsseldorf, Deutschland; 2 Bundeszentrale für gesundheitliche Aufklärung (BZgA), Köln, Deutschland

Erratum zu:

Bundesgesundheitsblatt - Gesundheitsforschung - Gesundheitsschutz 2021

10.1007/s00103-021-03304-1

In diesem Artikel fehlte die Lizenzangabe. Sie hätte lauten sollen: „Open Access. Dieser Artikel wird unter der Creative-Commons-Namensnennung‑4.0‑International-Lizenz veröffentlicht, welche die Nutzung, Vervielfältigung, Bearbeitung, Verbreitung und Wiedergabe in jeglichem Medium und Format erlaubt, sofern Sie den/die ursprünglichen Autor(en) und die Quelle ordnungsgemäß nennen, einen Link zur Creative-Commons-Lizenz beifügen und angeben, ob Änderungen vorgenommen wurden.

Die in diesem Artikel enthaltenen Bilder und sonstiges Drittmaterial unterliegen ebenfalls der genannten Creative-Commons-Lizenz, sofern sich aus der Abbildungslegende nichts anderes ergibt. Sofern das betreffende Material nicht unter der genannten Creative-Commons-Lizenz steht und die betreffende Handlung nicht nach gesetzlichen Vorschriften erlaubt ist, ist für die oben aufgeführten Weiterverwendungen des Materials die Einwilligung des jeweiligen Rechteinhabers einzuholen.

Weitere Details zur Lizenz entnehmen Sie bitte der Lizenzinformation auf http://creativecommons.org/licenses/by/4.0/deed.de.“

Der Originalbeitrag wurde korrigiert.

